# Analysis of the effectiveness of Sodium Hypochlorite decontamination of cadaveric human tissues at retrieval

**DOI:** 10.1007/s10561-016-9589-y

**Published:** 2016-10-18

**Authors:** Adolfo Paolin, Diletta Trojan, Antonio Carniato, Fabio Tasca, Ervino Massarin, Alessandro Tugnoli, Elisa Cogliati

**Affiliations:** Treviso Tissue Bank Foundation, Piazzale Ospedale 1, 31100 Treviso, Italy

**Keywords:** Sodium hypochlorite, Retrieval, Tissue banking, Homografts, Human tissues

## Abstract

Bacterial contamination of tissues retrieved from cadaveric donors is a common feature worldwide, and every tissue bank, albeit using different methods, conducts decontamination to guarantee safe tissues suitable for clinical use. The effectiveness of the methods used to eradicate pathogens differs. In order to reduce the tissue bioburden at retrieval, we have introduced a new method involving rinsing tissues in a sodium hypochlorite solution. To test its effectiveness we analyzed two comparable groups of tissues: Group A: 1881 tissues, all rinsed with isotonic saline solution after retrieval, and Group B: 1968 tissues immersed in an isotonic saline solution containing sodium hypochlorite (final concentration 0.1 %) for different lengths of time and subsequently rinsed with isotonic saline. The rinsing solution of each tissue was then sampled for microbiological cultures in both groups. The resultant overall contamination rate was 40.5 % for Group A and 6.7 % for Group B, with an 82.8 % difference in the reduction of contamination between the two groups. This was especially the case for commensal skin bacteria in musculoskeletal tissue, which accounted for over half the overall contamination. Our data highlighted that decontamination with sodium hypochlorite was helpful in reducing the bacterial bioburden in tissues retrieved from cadaveric donors.

## Introduction

Reducing the rate of homograft microbiological contamination at retrieval is a crucial aspect of tissue banking. Despite strict donor selection and tissue retrieval criteria, primary contamination of harvested tissues is frequently found. This leads to considerable rejection and expenditure for the microbiological tests needed to identify the pathogens and ascertain the sensitivity of the antibiotic cocktails normally employed for the next phase of decontamination. In order to reduce the bioburden we developed a sodium hypochlorite-based tissue decontamination procedure at retrieval. NaOCl is an antimicrobial agent applied in the clinical setting on account of its wide antimicrobial spectrum (Rutala and Weber 1997, Hidalgo et al. 2002). When coming into contact with organic tissues, hypochlorous acid (HOCl), a substance present in the NaOCl solution, releases chlorine that combines with the protein amino group to form chloramines, which in turn interfere with cell metabolism. Their antimicrobial effect is exerted by inhibiting essential bacterial enzymes through irreversible oxidation (Estrela et al. 2002). HOCl oxidation completely inhibits the active transport of energy sources for bacterial ATP synthesis, disrupts cytoplasmic membranes and interferes with phospholipid degradation (Barrette et al. 1989). Variously diluted, NaOCl is used for vaginal lavage, bladder and urethra irrigations, on burn infections (Bloomfield 1996) and as a root canal irrigant in dental surgery (Kuruvilla and Kamath 1998; Sen et al. 1999).

NaOCl has many properties of the ideal disinfectant, such as a broad antimicrobial spectrum, rapid bactericidal action, ease of use, solubility in water, stability in both its concentrated and diluted forms, non-toxicity to humans at normal usage concentrations, absence of poisonous residuals, colorless, nonflammable, and cheap (Rutala and Weber 1995). As an antiseptic, NaOCl is also used in many hospitals as a first choice to sanitize smooth surfaces. Still unclear, however, is the depth of NaOCl penetration into the tissues.

NaOCl decontaminates but has, only in low concentrations, no significant effects on cell viability (Cotter et al. 1985). Used as a first-step procedure to reduce primary contamination of tissues retrieved from cadaveric donors cuts the number of microbiological seedings and pathogen identifications required when microbiological cultures prove positive. Accordingly, the aim of our study was to verify the efficacy of our protocol of rinsing tissues with a low concentration of NaOCl-containing solution to reduce the bioburden at retrieval.

## Materials and methods

### Tissue sample and decontamination protocol

The musculoskeletal (MST) and cardiovascular tissues (CVT) retrieved from 344 cadaveric donors over two consecutive years were included in this study. Tissue retrieval was performed within 24 h of cardiac arrest by our retrieval team of physicians and technicians. Prior to tissue retrieval, the skin underwent surgical scrubbing with a chlorhexidine solution and shaving, with an additional application of chlorhexidine and povidone iodine. The whole procedure was conducted in the operating theater.

Tissues were divided in two groups: Group A, donors whose tissues were not treated according to the decontamination protocol, and Group B, donors whose tissues were decontaminated according to our protocol. The two groups were comparable in terms of donor characteristics and type and number of tissues considered (see Tables 1, 2). Decontamination of Group B tissues was carried out immediately after retrieval as follows: musculoskeletal tissues (MST) were immersed in an isotonic saline solution containing 0.1 % sodium hypochlorite for 5 min and cardiovascular tissues (CVT) for 3 min, and rinsed with isotonic saline solution. Tissues were shaken both when immersed in the decontaminating solution and subsequently in the rinsing solution and all these procedures were carried out at room temperature. Cardiac valves were not included in the CVT since they are extracted the day after retrieval. Group A tissues were just rinsed with isotonic saline solution after retrieval. The rinsing solution of each tissue was then sampled without filtering for microbiological cultures in both groups.

**Table 1:** Donor characteristics data

	Group A	Group B
Heart-Beating	38	24
Non-Heart-Beating	136	146
Male	137	135
Female	37	35
Mean age (years)	43,6	44,9
Total	174	170

**Table 2:** Tissues data

Tissue type	N° of tissues retrieved	
	Group A	Group B
MST		
Single bones	1694	1783
Costal cartilage/ribs		
Fascia lata		
Femoral condyles, hemicondyles		
Femoral diaphysis, heads, struts		
Tendons		
Iliac crest		
Knee		
Menisci with tibial plateau		
Tibial diaphysis		
CVT		
Abdominal aorta	187	185
Aortic conduit		
Arterial and venous segments		
Heart with or without thoracic aorta		
Pericardium		
Total	1881	1968

Once in the bank, retrieved tissues underwent two-pass decontamination with an antibiotic cocktail, before processing and before freezing, followed by two microbiological controls. In compliance with our policy, we have classified some strains such as *Clostridium spp., Fungi/Yeast, Mycobacteria, Streptococcus pyogenes, Streptococcus pneumoniae, Pseudomonas aeruginosa, Serratia marcescens, Meningococcus* as non-compliers; if any of these were isolated the tissue was discarded regardless of the step at which positivity was detected. In addition to discarding tissues contaminated with non-compliers, all tissues found to be positive after the 2nd decontamination were also discarded.

### Microbiological analysis

Microbiological cultures were carried out using BD BACTEC Fluorescent Test Technology (BM BACTEC™ plus aerobic/F and anaerobic/F culture vials). Culture bottles were incubated at 36.5 °C for 7 days. Each vial contains a chemical sensor able to detect increases in CO_2_ produced by the growth of microorganisms and fluorescence, subsequently monitored by a BACTEC fluorescent series instrument. Culture bottles showing evidence of growth after 7 days were gram stained, subcultured on blood agar plates and incubated for 48 h at 35/38 °C in a normal atmosphere, an enriched atmosphere of 5 % CO_2_, and an anaerobic atmosphere. Samples were then processed under a Biohazard Class II laminar flow hood and all bacteria identified with the standard biochemical procedure. Lastly, an antibiogram was drawn up for each bacterium isolated and Minimal Inhibitory Concentration estimation performed in the standard media used in clinical practice. Samples were also cultivated in a Lowenstein-Jensen medium to isolate mycobacteria. Microbiological cultures and analyses were carried out by an accredited in-hospital Microbiology laboratory and interpreted by a microbiologist with specific expertise.

### Tissue evaluation

Every tissue was assigned a score on a 5-point scale (1 = worst, 5 = best). Scores were determined on the morphological and mechanical characteristics of the tissues as assessed by two examiners at different times: post-processing and before release. CVT morphological assessment consists of visual and manual inspection, while structural integrity is ascertained with a pressure test. MST tissue is assessed in the same way, with, if necessary, dynamic-mechanical testing to verify MST mechanical properties. In line with our internal policy, tissues with scores from 1 to 3 were discarded, and only those with scores of 4 and 5 were retained for clinical use. Tissue discard rates due to morphological changes were recorded and compared between the two groups. In addition, any report of adverse event/reaction attributable to tissue implantation was reported on our standard report form.

## Results

### Microbiological results

Overall microbiological findings are summarized in Table 3. In Group A, 762 of the total 1881 tissues were found to be contaminated while in Group B, 131 out of 1968 proved positive. The total contamination percentage was 40.5 % in Group A and 6.7 % in Group B, with a difference in contamination rate reduction between Groups A and B of some 83 %. More specifically, in Group A 627 MST (37.0 % of total MST), and 135 CVT (72.1 % of total CVT) were contaminated at culture, while 29 MST (1.6 % of total MST) and 102 CVT (55.1 % of total CVT) in Group B were positive, with decontamination rate differences of 95.3 % and 24.4 % in the MST and CVT respectively between Groups A and B.

**Table 3:** Number of tissues retrieved and contaminated in Group A and Group B, with relative percentages (%)

Tissue type	Group A			Group B			Comparison
	N° of tissues retrieved	N° of contaminated tissues	Contamination rate (%)	N° of tissues retrieved	N° of contaminated tissues	Contamination rate (%)	Group A vs Group B % decrease in contamination (%)
MST	1694	627	37	1783	29	1.6	−95.3
CVT	187	135	72	185	102	55	−24.4
TOTAL	1881	762	40.5	1968	131	6.7	−83

A total of 872 strains were isolated (1.1 strains per tissue) in Group A and a total of 169 (1.3 strains per tissue) in Group B, of which 26 and 18 genera were identified in Group A and B respectively. In Group A, coagulase-negative *Staphylococc*i accounted for over half the positive cultures (71 %), *Streptococcus* for 9.6 *%, Staphylococcus aureus* for 3.7 % and *Clostridium* for 3.3 %. The other 22 genera accounted for the remaining 12.4 % contaminations). In Group B, the pathogens most frequently isolated were: coagulase-negative *Staphylococci* (36.1 % of total), *Streptococcus spp* (16.6 % of total), *Clostridium spp* (13 % of total), *Bacillus spp* (8.2 % of total) and *Escherichia coli* (5.9 % of total). The 14 other genera accounted for the remaining 20.2 % of the contaminated tissues. No Mycobacteria were isolated in either group. (Table 4).

**Table 4:** Genera and n° of strains isolated in Group A and Group B

Genera	N° of strains	%
Group A		
*Staphylococcus coagulase* – *spp*	619	71.0
*Streptococcus spp*	84	9.6
*Staphylococcus aureus*	32	3,7
*Clostridium spp*	29	3,3
*Enterococcus spp*	26	3,0
*Micrococcus luteus*	13	1.5
*Bacillus spp*	9	1.0
*Granulicatella adiacens*	8	1.0
*Bacteroides spp*	7	0.8
*Escherichia coli*	7	0.8
*Klebsiella spp*	7	0.8
*Corynebacterium propinquum*	5	0.6
*Stenotrophomonas maltophilia*	5	0.6
*Peptostreptococcus spp*	4	0.5
*Leuconostoc mesenteroides*	3	0.4
*Brevibacterium spp*	2	0.2
*Kocuria kristinae*	2	0.2
*Serratia spp*	2	0.2
*Acinetobacter baumanii*	1	0.1
*Aeromonas hydrophyla*	1	0.1
*Aerococcus viridans*	1	0.1
*Chryseobacterium indologenes*	1	0.1
*Haemophilus influenzae*	1	0.1
*Lactococcus lactis*	1	0.1
*Pseudomonas aureginosa*	1	0.1
*Sphingomonas paucimobilis*	1	0.1
Total	872	
Group B		
*Staphylococcus coagulase* - *spp*	61	36.1
*Streptococcus spp*	28	16.6
*Clostridium spp*	22	13.0
*Bacillus spp*	14	8.2
*Escherichia coli*	10	5.9
*Staphylococcus aureus*	9	5.3
*Klebsiella spp*	5	3.0
*Peptostreptococcus spp*	4	2.3
*Bacteroides spp*	3	1.8
*Aerococcus viridans*	2	1.2
*Gemella morbillorum*	2	1.2
*Micrococcus luteus*	2	1.2
*Proteus spp*	2	1.2
*Aeromonas hydrophila*	1	0.6
*Aspergillus niger*	1	0.6
*Enterecoccus faecalis*	1	0.6
*Propionibacterium acnes*	1	0.6
*Sphingomonas paucimobilis*	1	0.6
Total	169	

Isolated strains per tissue are presented in Table 5 for both groups. The decontamination procedure led to a fall in the number of positive MST: from 675 in Group A to 31 in Group B (−95.4 %), in the number of genera, from 22 to 8, and a reduction in the number of positive CVT cultures: from 197 in Group A to 138 in Group B (−30 %) and in genera, from 17 to 16 respectively. In Group A, each MST was observed to contain 1.0 strain while CVT contained 1.4 strains each. In Group B, each MST sample contained 1.0 strain while CVT were observed to have 1.3 strains each. The ratio of strains isolated per tissue remained unchanged both comparing the groups and tissue types. Of the main strains found in MST, *Staphylococcus coag* spp. fell by 98 %, *Streptococcus * spp. by 96,4 %, while *Staphyilococcus aureus, Enterococcus spp* and *Micrococcus luteus* were completely eradicated. In CVT tissues, *Staphylococcus coag* spp. fell by 26.5 %, *Streptococcus spp* by 51.2 %, and *Clostridium spp* by 31.8 %, while *Staphylococcus aureus* remained unchanged.

**Table 5:** Genera and n° of strains isolated in MST and CVT  in Group A and B

Genera	N° of strains	
	Group A	Group B
MST		
*Staphylococcus coag* - *spp*	551	11
*Streptococcus spp*	28	1
*Staphylococcus aureus*	23	–
*Enterococcus spp*	17	–
*Micrococcus luteus*	12	–
*Bacillus spp*	8	7
*Clostridium spp*	7	7
*Corynebacterium propinquum*	5	–
*Bacteroides spp*	4	–
*Escherichia coli*	4	2
*Brevibacterium spp*	2	–
*Granulicatella adiacens*	2	–
*Kocuria kristinae*	2	–
*Serratia spp*	2	–
*Acinetobacter baumanii*	1	–
*Aereomonas hydrophyla*	1	1
*Chryseobacterium indologenes*	1	–
*Klebsiella spp*	1	–
*Peptostreptococcus spp*	1	1
*Pseudomonas aureginosa*	1	–
*Sphingomonas paucimobilis*	1	–
*Stenotrophomonas maltophila*	1	–
*Aspergillus niger*	–	1
Total	675	31
CVT		
*Staphylococcus coag* – *spp*	68	50
*Streptococcus spp*	56	27
*Clostridium spp*	22	15
*Staphylococcus aureus*	9	9
*Enterococcus faecalis*	9	1
*Granulicatella adiacens*	6	–
*Klebsiella spp*	6	5
*Stenotrophomonas maltophila*	4	–
*Bacteroides spp*	3	3
*Escherichia coli*	3	8
*Leuconostoc mesenteroides*	3	–
*Peptostreptococcus spp*	3	3
*Aerococcus viridans*	1	2
*Bacillus spp*	1	7
*Haemophilus influenzae*	1	–
*Lactococcus lactis*	1	–
*Micrococcus luteus*	1	2
*Gemella morbillorum*	–	2
*Propionibacterium acnes*	–	1
*Proteus spp*	–	2
*Sphingomonas paucimobilis*	–	1
Total	197	138

### Tissue evaluation

The percentage of tissues discarded in both Groups on account of morphological changes was comparable for both tissue types: 7.5 and 8.2 % MST, and 53 and 57.6 % CVT in Groups A and B respectively. Moreover, no adverse event/reaction attributable to the tissues was reported by end users subsequent to implantation.

## Discussion

The survey highlighted clearly that first-step decontamination with sodium hypochlorite is a useful adjuvant treatment to reduce bacterial bioburden in tissues retrieved from cadaveric donors.

Since there was no previous report in the literature on decontamination of human tissue with NaOCl, and given the fact that our end-point was bioburden reduction, and not the complete eradication of all contamination, we decided to start with the lowest concentration and exposure time indicated in the experimental study by Cotter et al. al. who started with concentrations of 0.1 % NaOCl and exposures of 5 min, proceeding to assess the efficacy of increasing concentrations and exposure times (Cotter et al. 1985) in decontamination of cadaveric skin. They found NaOCl to have antimicrobial activity already at the lowest concentration and exposure time. Since it was observed during the protocol development phase that after the established 5-minute exposure the outer surface of the CVT tissues had become opaque, it was decided to reduce the exposure time to 3 min, a duration that did not cause tissue opacification, for fear that the change in appearance might signal structural damage.

In our analysis, coagulase-negative *staphylococci* were the most frequently isolated strains both in MST and CVT, although found in a much higher percentage in MST than CVT. A higher percentage of intestinal bacteria was frequently detected in CVT. Since our donors presented no systemic or local infections at the time of screening, and since harvesting was conducted in the operating theater by specialist personnel using sterile equipment, it may be argued that tissue contamination was caused by other factors. The presence of skin commensals, the most frequently isolated germs in our sample, is probably the result of external contamination at the time of procurement due to leakage from the skin incisions made to access the thoracic and abdominal cavities, and of exposure to the environment during retrieval and/or handling. Conversely, the high percentage of intestinal and upper airway bacterial strains in CVT specimens may have multiple causes, the most important being the actual cause of donor death, which in more than 50 % of our sample was trauma (paper in preparation). Trauma exponentially increases the risk of contamination by intestinal and upper airway bacteria, as confirmed by Deijkers et al., who found that the risk of graft contamination with highly pathogenic organisms increased by a factor of 3.4 in the case of donor death by trauma (Deijkers et al. 1997). The same explanation is also given by Martinez et al., who argue that seeding of the bloodstream could develop in victims of extensive trauma or following invasive procedures during emergency care, as in the case of road accident victims or cardiac infarction patients (Martinez et al. 1985). CVT is often collected from the thoracic and abdominal cavities of donors who have suffered trauma-induced hemorrhagic effusion that may facilitate passive and active cross-contamination. In addition, donors are often removed from the place of death, be it street or home, after several hours and prolonged warm ischemic time, which facilitates the growth and migration of bacteria into the bloodstream prior to transportation and refrigeration in the referring hospital morgue. Van Kats has also confirmed a significant relationship between warm ischemic time and contamination at retrieval (Van Kats et al. 2010).

Despite differences among the bacterial strains isolated in the tissues, decontamination nonetheless proved effective in both tissue types. In short, the decontamination procedure almost completely eradicated the 5 pathogens most frequently detected in MST, while decontamination was much less effective on the 5 pathogens most frequently found in CVT, a striking finding, considering that the same three most frequently identified pathogens were found in both MST and CVT. Decontamination efficacy remained low or absent for other strains, which, however, presented less frequently in both groups. Of note is the fact that the pathogens found in CVT specimens are prevalently endogenous in nature and that the average number of strains isolated was >1, a finding that remained largely unchanged even after the decontamination procedure, showing the only moderate decontamination effect achieved on CVT compared to MST.

The only difference between the two types of tissues was decontamination time: 5 min. for MST and 3 min. for CVT. Cotter et al. decontaminated samples of human cadaveric skin colonized by *S. aureus, Pseudomonas aeruginosa,* or *Candida albicans* in vitro with a freshly prepared 0.1 % NaOCl solution within 5, 10, 20, and 30 min of exposure, respectively, demonstrating that extending the tissue exposure time to the decontamination solution totally eradicated pathogens, including spore-forming germs, fungi and yeasts (Cotter et al. 1985). In light of these findings, it may be reasonably assumed that the lower decontamination efficiency found by us in CVT is due to the shorter duration of this tissue type in the NaOCl solution. This hypothesis is further corroborated by the fact that the strains most frequently identified in CVT did not substantially differ from skin commensals and strains originating from endogenous sources like upper respiratory or gastrointestinal tract and that, in conditions of equal sensitivity, the length of time in the NaOCl solution affects the extent of decontamination. While concentrations of less than 0.025 % have been found to be non-bactericidal for Gram- organisms with variable intra- and inter-species inhibition at 5-, 10-, 15-, and 30 min intervals (Heggers et al. 1991), and not induce cell death in vitro (Kozol et al. 1988) after 30 min of exposure, Hidalgo et al. have shown that concentrations >0.05 % have a complete cytotoxic effect with 100 % mortality at all exposure times assayed (varying between 2 and 24 h) (Hidalgo et al. 2002). Missotten et al. have demonstrated that no surviving ocular cells were observed after treatment for 3 min with 0.5 % NaOCl in vitro (Missotten et al. 2008). Concentrated hypochlorite solutions are known to produce intra-clinical damage to intact skin (Goffin et al.^1997^) while 15-min exposure of cultured fibroblasts to diluted solutions (NaOCl 0.5 %) has been reported to cause total cell destruction (Lineaweaver et al. 1985). Most of the cell decay occurs within the first 2–5 min of the exposure (Barrette et al. 1989) with the consequent irreversible destruction of cell’s energy-producing capability. NaOCl in lower concentrations (0.5–1 %) has been found to be biocompatible (Holland et al. 1992) i.e. present moderate or intense tissue reaction at 7 days, which decreases in intensity with time until no significant tissue reaction is registered (Costa 2001). Any loss of cellularity by tissues following immersion in solutions containing low concentrations of NaOCl does not jeopardize their subsequent clinical use. In fact it is well known that once retrieved, tissues lose almost all their original cellularity, functioning in the body of the recipient mainly as scaffolds to be repopulated with host cells. It is still unclear if as well as cell death, NaOCl induces structure-altering ECM lesions. We carefully examined tissue quality to identify any modification or change in morphology or mechanical strength in the tissues undergoing the decontamination protocol and found no major visible morphological difference in the tissues of Group A and Group B. In fact, the total percentage of discarded tissue in the first year of the protocol’s application did not differ from the control year, either for MST or CVT. Furthermore, even if tissue assessment was qualitative, there were no subsequent reports of tissue unsuitability or of any adverse event following implantation of the NaOCl-treated tissues in the forms filled in by end-users.

NaOCl in solution is an attractive way of decontamination of cadaveric tissues for many reasons: it is easily available on the market, inexpensive, and may be prepared on the spot in the operating theater before tissue retrieval. Its use was seen to dramatically cut laboratory costs due to the hundreds of microbiological analyses required to identify bacteria during the first year of protocol implementation. Last but not least, treatment efficacy was not associated in our samples with any evident changes in tissue quality. Numerous sterilants and sterilant combinations are used to eradicate microorganisms on allografts. A survey of orthopedic surgeons, however, found that 81 % believed that tissue quality was to some extent compromised by sterilization processes (McAllister et al. 2007). Guaranteeing a Sterility Assurance Level (load < 10^−6^) requires irradiation dose greater than 20–25 kGy, which induces major changes in the extracellular matrix. This method would in no way be feasible for CVT. Our tissue bank does, however, freeze-dry MST and perform terminal sterilization and virus inactivation with 25 kGy irradiation according to a validated procedure. This is performed exclusively on bone tissues destined for use as chips, bone segments and putty. When terminal irradiation is carried out, less stringent harvesting and processing safety standards may be applied and antibiotic decontamination procedures omitted since practically all tissues will be retained. If, as in our case, tissues are not irradiated but first rinsed in NaOCl for a few minutes and then decontaminated in antibiotic throughout the processing phase, the end result will be safe, less damaged tissue, which however, carries a higher risk of having to be discarded at the end of the process.

Specific studies are, however, needed to verify the hypothesis that increased concentrations or exposures to NaOCl really can eradicate all pathogens without leading to tissue structural damage that would jeopardize their clinical use. If our hypothesis were to be proved correct, then NaOCl solution decontamination might replace the final antibiotic decontamination adopted by most tissue banks in the post-processing phase, a change that would be both practically and economically advantageous.
